# Genomic and Functional Characterization of Vancomycin-Resistant Enterococci-Specific Bacteriophages in the *Galleria mellonella* Wax Moth Larvae Model

**DOI:** 10.3390/pharmaceutics14081591

**Published:** 2022-07-30

**Authors:** Lynn El Haddad, Georgios Angelidakis, Justin R. Clark, Jesus F. Mendoza, Austen L. Terwilliger, Christopher P. Chaftari, Mark Duna, Serena T. Yusuf, Cynthia P. Harb, Mark Stibich, Anthony Maresso, Roy F. Chemaly

**Affiliations:** 1 Department of Medicine, University of Florida, Gainesville, FL 32611, USA; lynnelhaddad@ufl.edu (L.E.H.); jesus.barreramendoza@medicine.ufl.edu (J.F.M.); 2 Department of Infectious Diseases, Infection Control and Employee Health, The University of Texas MD Anderson Cancer Center, Houston, TX 77030, USA; gangelidakis@mdanderson.org (G.A.); cpchaftari@tamu.edu (C.P.C.); mduna2@mdanderson.org (M.D.); serena_yusuf@tamu.edu (S.T.Y.); cpharb@mdanderson.org (C.P.H.); mark.stibich@xenex.com (M.S.); 3 Department of Molecular Virology and Microbiology, Baylor College of Medicine, Houston, TX 77030, USA; justin.clark@bcm.edu (J.R.C.); terwilli@bcm.edu (A.L.T.); maresso@bcm.edu (A.M.); 4 Xenex Disinfection Services, San Antonio, TX 78216, USA

**Keywords:** bacteriophage, *Galleria mellonella*, vancomycin-resistant *Enterococcus faecium*

## Abstract

Phages are naturally occurring viruses that selectively kill bacterial species without disturbing the individual’s normal flora, averting the collateral damage of antimicrobial usage. The safety and the effectiveness of phages have been mainly confirmed in the food industry as well as in animal models. In this study, we report on the successful isolation of phages specific to Vancomycin-resistant Enterococci, including *Enterococcus faecium* (VRE*fm*) and *Enterococcus faecalis* from sewage samples, and demonstrate their efficacy and safety for VRE*fm* infection in the greater wax moth *Galleria mellonella* model. No virulence-associated genes, antibiotic resistance genes or integrases were detected in the phages’ genomes, rendering them safe to be used in an in vivo model. Phages may be considered as potential agents for therapy for bacterial infections secondary to multidrug-resistant organisms such as VRE*fm*.

## 1. Introduction

By some estimates, infections from multidrug-resistant organisms (MDROs) will cause 10 million deaths per year by 2050 worldwide—more deaths than cancer [1]. Despite the efficacy of antibiotics to prevent or treat bacterial infections, their long-term use is associated with many sequelae, including the development of MDROs and the disruption of the microbiota, the gut microbiota, in particular, which may lead to the translocation of bacteria, including MDROs, into the bloodstream with a subsequent increase in complications and mortality [2]. Vancomycin-resistant *Enterococcus faecium* (VRE*fm*) is a major and prevalent MDRO with considerable clinical infection control and public health implications [3,4]. The ability of VRE*fm* clones to transfer genes encoding resistance to drugs such as vancomycin and daptomycin to other bacteria [5], their adaptation to harsh environments, and their long-term survival on high-touch surface areas contribute to an increased risk of VRE*fm* colonization and transmission to other patients through the environment, the hands of healthcare workers, and/or equipment [6,7,8,9]. Particularly, VRE*fm* colonization in the gastrointestinal tract can also lead to VRE*fm* infection in high-risk individuals, such as cancer patients [2,4,10]. VRE*fm* dominance in the gut can occur when the gut microbiome is disturbed and does not recover completely. It is hypothesized that the loss of some bacterial populations after antibiotic therapy and the disruption of the normal flora enables the expansion of VRE*fm* and its dominance of the gut microbiome [11], leading to the loss of heterogeneity of the gut microbiome and an increased rate of morbidity and mortality in these patients [11,12].

Considering the public health concerns related to MDROs in healthcare settings and the lack of accepted and effective interventions for their eradication, new strategies to prevent or treat potential MDRO infections, thus improving clinical outcomes, are needed. Bacteriophages (or phages) are naturally occurring viruses that kill selective bacterial species. They are the most abundant organisms in the biosphere with an estimated number of 10^31^ phage particles [13]. Phages may constitute a good or adjunct alternative to antibiotics because of their (1) specificity to prevent or control specific bacterial species without disrupting the host’s microbiome and averting other drawbacks of antimicrobial usage [13,14,15], (2) co-evolution with the bacterial host that limits resistance, (3) safety and lack of side effects for humans [16,17], and (4) cost-effectiveness in phage production for large-scale applications [18]. Several studies have been reported in the United States on the safety and efficacy of phages and no known significant adverse events have been detected in healthy immunocompetent individuals or immunocompromised patients [19,20,21,22,23]. We recently reviewed the studies involving phage therapy against MDROs in a clinical setting [22]. These studies demonstrated good phage efficacy and safety.

In this study, we isolate VRE*fm* phages from wastewater to design effective combinations against prevalent VRE*fm* strains isolated in the hospital environment. Furthermore, we use the *Galleria mellonella* or the greater wax moth larva model as an in vivo pre-screening model, preceding the mammalian model, to study the safety and efficacy of bacteriophages in eradicating the predominant strains of VRE*fm*. This host is an alternative and innovative model to study microbial virulence, as well as to evaluate the efficacy of antimicrobial agents such as antibiotics and phage therapy [24,25].

## 2. Materials and Methods

### 2.1. Bacterial Strains and Media

A vancomycin-susceptible enterococci (VSE) was used to isolate phages from wastewater samples. Phages were then propagated on several VRE*fm* strains isolated from the hospital environment, rectal swabs from cancer patients, and wastewater samples, along with the VSE strain and an *Enterococcus faecalis* strain purchased from the Félix d’Hérelle collection (www.phages.ulaval.ca, accessed on 20 July 2022). Tryptic Soy Broth (TSB, Becton Dickinson) was used for bacterial culture and phage amplification.

### 2.2. Phage Recovery

A wastewater sample was obtained from a local municipal facility in Houston, Texas. The sample was centrifuged at 10,000× *g* for 10 min and filtered using a 0.45 µm syringe filter. The phage isolation procedure is reported elsewhere [26]. Briefly, an overnight culture of 100 µL of VSE was added to 5 mL of 2 times concentrated TSB and 5 mL of the filtered sewage sample, and incubated overnight at 37 °C as a first amplification. After several amplifications, the final filtrate was checked for the presence of VRE*fm*-specific phages by a spot test [26,27]. Several clear phage plaques were picked, purified, and characterized. 

### 2.3. Host Range

The recovered VRE*fm* phages were first characterized by determining their host ranges on a panel of 12 strains of enterococci isolated from different sources: stool samples from VRE*fm*-colonized patients and VRE*fm*-infected patients, patient room environments, sewage samples, clinical isolates of daptomycin-resistant VRE*fm* strains, VSE, and *Enterococcus faecalis* strains [28].

### 2.4. Electron Microscopy

A 1.5 mL sample of phage lysate (titer of at least 10^9^ PFU/mL) was centrifuged at 23,500× *g* for 1 h at 4 °C. The supernatant was removed, leaving approximately 100 µL in the tube. The phage pellet was washed twice with 1.4 mL of ammonium acetate (0.1 M, pH 7.5). The residual volume (100 µL) was used to prepare the observation grid as follows: A 400-mesh carbon-coated Formvar nickel grid was glow-discharged using the PELCO easiGlow (Ted Pella, Redding, CA, USA) followed by floating onto a 10 µL droplet of provided sample for 5 min. The sample grid was then floated on a 10 µL droplet of 2% aqueous phosphotungstic acid (pH 7.0) for 30 s and examined with a FEI Tecnai G2 Spirit Twin TEM (FEI Corp., Hillsboro, OR, USA). The residual liquid was removed from the grid by touching the edge with blotting paper. Digital images were acquired with a Gatan UltraScan 1000 2k × 2k camera and Digital Micrograph software (Gatan Inc., Pleasanton, CA, USA). Phages were observed at 120 kV using a Tecnai G2 Spirit TWIN transmission electron microscope (200 nm) located at the Electron Microscopy Core of the University of Florida’s Interdisciplinary Center for Biotechnology Research [29,30].

### 2.5. Phage DNA Preparation and Sequencing

DNA extraction was performed on the selected phage lysates using the Phage DNA isolation kit (NORGEN Biotek) according to the manufacturer’s instructions with DNase/Rnase treatment [31]. Genome sequencing was performed using an Illumina MiSeq with 250 bp paired-end reads. The extracted DNA was further cleaned up using a QiaQuick PCR purification kit as per the manufacturer’s instructions (Qiagen). The library was prepared using a Nextera Flex kit followed by sequencing on a 250 PE run on the Illumina MiSeq [32].

### 2.6. Bioinformatic Analysis

Raw reads were processed using FaQCs [33] and assembled using the Geneious assembler [34,35]. For each phage, a single contig produced the final assembly. Gene calling and annotation were performed using the RASTtk [34] pipeline through PATRIC’s Genome Annotation service [35] and tRNA predictions were completed using ARAGORN [36]. Coding sequences (CDS) and reads were searched for virulence and antibiotic resistance genes by using BLAST [37] to compare assembled genomes against the Virulence Factor Database (VFDB) [38], the PATRIC Virulence Factor Database [39], the Antibiotic Resistance Gene Database (ARDB) [40], and the Comprehensive Antibiotic Resistance Database (CARD) [41]. ShortBRED [42] was used for targeted searches of coding sequences and reads for genes in VRDB, CARD, and the Resfam Antibiotic Resistance Gene Database through EDGE Bioinformatic [43,44]. Phage genus was predicted from the sequenced relatives identified by using BWA-Mem (version 0.7.9) [45] by aligning contigs to NCBI’s RefSeq database and by CDS homology using Phage Search Tool Enhanced Release (PHASTER) [46]. Phage lifestyles were predicted using PHACTs [47]. Integrases and attachment sites were searched for using PHASTER and by parsing annotated genomes for “integrase.” The percentage of total reads mapped to the host was determined by aligning reads to NCBI’s RefSeq database using BWA-Mem and determining the number of reads that mapped to an *Enterococcus faecalis* genome over the total number of reads. Alignments were produced using Mauve (version 2.4.0) [48].

### 2.7. In Vivo Efficacy of the Phages

Isolated and characterized phages were combined in a phage cocktail or phage mixture and tested for their efficacy and safety in an in vivo model. *Galleria mellonella* larvae were injected with VRE004, a VRE*fm* strain isolated from stools of a VRE-infected HCT recipient at a concentration of 10^7^ colony-forming units (CFU)/10 µL. Two isolated phages were combined in a cocktail and injected at a concentration of 2 × 10^6^ plaque-forming units (PFU)/10 µL 1 h post-bacterial injection. An additional group of larvae received the same phage cocktail 1 h prior to VRE*fm* injection as a prevention or prophylactic group. Control groups included larvae injected with bacteria alone, phages alone (to measure toxicity due to phage administration), sterile medium (to measure any lethal effects due to physical trauma from the injection), or without any manipulation [49]. Each group comprised 8 larvae and the experiments were replicated 5 times. The insect’s health state and mortality were observed and scored after 24 h and 48 h of incubation at 37 °C using a published health index scoring system [50]. Using STATA version 12, we conducted two mixed-effect logistic regression models, with conditions predicting the number of surviving larvae by 48 h of follow-up and controlling for replicates. For the first model, we compared a saline solution (control) to phages alone. For the second model, we compared VRE*fm* alone to VRE*fm* with phages. Finally, we conducted a one-way analysis of variance to compare VRE*fm* alone to the presence of the phage cocktail and to the control group with respect to VRE*fm* abundance. For each relevant statistically significant finding, we reported the *p*-value with 95% confidence intervals.

## 3. Results

### 3.1. Characterization of the Isolated Phages

**Figure 1:**
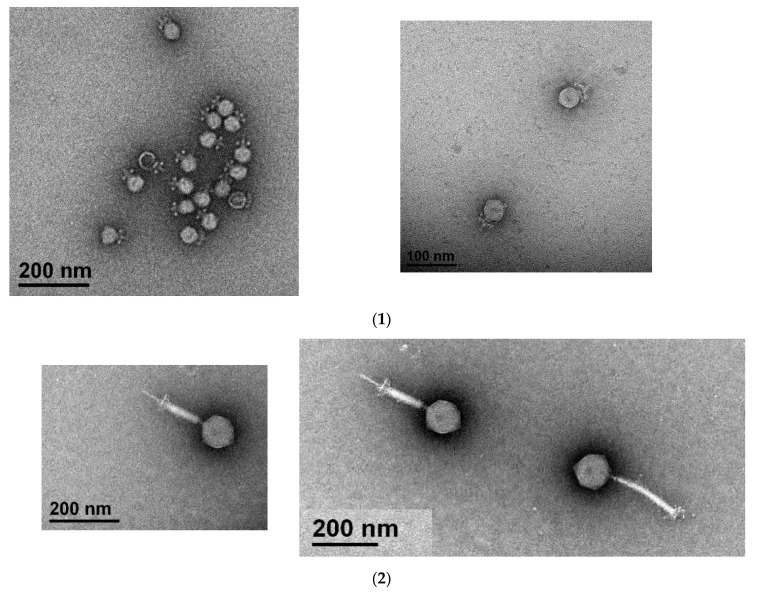
Visualization of phages MDA1 (**1**) and MDA2 (**2**) under the electron microscope. Scale bars indicate 100 nm and 200 nm.

Two enterococci phages (MDA1 and MDA2) targeting several VRE*fm* and VSE strains were successfully isolated. Both phages belonged to the *Caudovirales* order. Phage MDA1 belongs to the *Podoviridae* family, whereas phage MDA2 belongs to the *Myoviridae* family. *Podoviridae* and *Myoviridae* can be mainly distinguished by the length of their tails; *Podoviridae* are characterized by their short tail and *Myoviridae* have a long contractile tail [29]. Phage MDA1 has an icosahedral head of 40.4 ± 1.4 nm in diameter and a non-contractile tail with a length of 18.6 ± 1.6 nm. Phage MDA2 has an icosahedral capsid of 100.8 ± 7.2 nm in diameter, and a contractile tail of 179 ± 25 nm in length and 17.2 ± 5.1 nm in width (Figure 1). Both phages were lytic and were able to eradicate at least 6 out of the 12 tested VRE*fm* strains. The *Podoviridae* phages had a wider host range, infecting a total of 11/12 bacterial strains compared to 6/12 bacterial strains with the *Myoviridae* phages. The phage cocktail was able to eradicate all the 12 tested bacterial strains, including the daptomycin-resistant VRE*fm* strains (Table 1).

**Figure 2:**
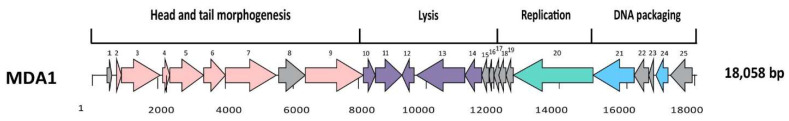
**Genome structure of MDA1 phage.** Each arrow represents an ORF. Colors represent different genomic regions including the DNA packaging (blue), the head and tail morphogenesis genes (pink), host lysis genes (purple), and the replication–transcription region (green). Grey arrows represent genes coding for hypothetical proteins.

**Figure 3:**
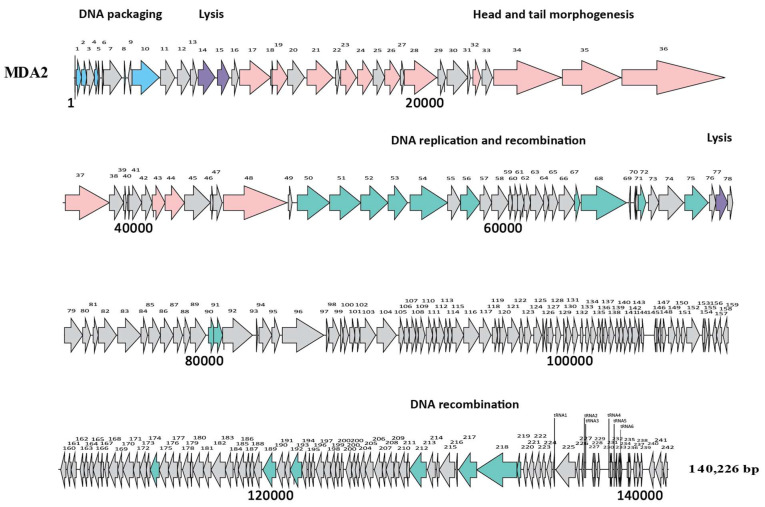
**Genome structure of MDA2 phage.** Each arrow represents an ORF. Colors represent different genomic regions including the DNA packaging (blue), the head and tail morphogenesis genes (pink), host lysis genes (purple), and the replication–transcription region (green). Grey arrows represent genes coding for hypothetical proteins. tRNAs are represented by vertical lines.

The genomes of MDA1 and MDA2 were 18,058 bp and 140,226 bp long, respectively. MDA1’s linear genome had a GC content of 33% (Figure 2). No tRNA was found in the genome. When compared to other published enterococcal genomes, phage MDA1’s genome had 82% homology with Enterococcus phage vB_Efae230P-4 (Figure S1) [51]. Phage MDA2 had a circular genome with a GC content of 35.8%, which was similar to that of other enterococcal phages and of its enterococcal hosts (38%) [51,52,53]. Six tRNAs were found in its genome. tRNAs are usually present in the phage genome as a result of variance in codon usage between their genome and their host’s genome, enabling phages to bypass codons overused in the phage genes and compensate for the extra bias [54,55]. The linearized genome of phage MDA2 is represented in Figure 3. When compared to other published enterococcal genomes, the genome of MDA2 was 70%, similar to that of phage phiEF24C [56]. Both *Podoviridae* vB_Efae230P-4 and *Myoviridae* phiEF24C were isolated from the environment in Japan and targeted vancomycin-resistant *E. faecalis* [51,56] (Figure S2). Of interest, no virulence-associated genes, antibiotic resistance genes or integrase genes were detected in the phages’ genomes (Tables S1 and S2).

### 3.2. Safety and Efficacy of the Phage Cocktail

The phage cocktail (MDA1 and MDA2) was administered to VRE-injected larvae and survival was monitored for 48 h. At 48 h, larvae injected with saline only (control) were just as likely to be alive as larvae injected with the phage cocktail only (82.5% vs. 85% alive; OR = 1.2, SE = 0.8, *p* = 0.75) demonstrating the safety of the phages. Additionally, by 48 h of follow-up, larvae injected with VRE*fm* (including daptomycin-resistant VRE*fm*) and phages were 3.7 (treatment group) and 6.5 times (prophylactic group) more likely to survive than larvae injected with VRE*fm* only (55% vs. 25% alive; OR = 3.7, SE = 1.8, *p* = 0.07 and 67.5% vs. 25% alive; OR = 6.5, SE = 3.3, *p* < 0.001, respectively), demonstrating the efficacy of phages against VRE*fm* in a larva model. Then, 16S analysis was performed on all the larvae to qualitatively identify VRE*fm* abundance in each group. Larvae from the same group were pooled and homogenized with sterile media and sent for DNA extraction and 16S sequencing and analysis. The larvae groups that received sterile media or phages alone did not have any VRE*fm* and were comparable to the control (without any manipulation). VRE*fm* abundance was significantly higher in the group receiving VRE*fm* alone compared to the control (*p* < 0.001), the group receiving VRE*fm* and then phages (*p* < 0.001), or the group receiving phages and then VRE*fm* (*p* < 0.001). As expected, there were no significant differences in VRE*fm* abundance between the control, the group receiving VRE*fm* and then phages, or the group receiving phages and then VRE*fm*.

### 3.3. Data Availability

The genome sequences of MDA1 and MDA2 have been deposited in GenBank under accession numbers MW623430 and MW633168, respectively.

## 4. Discussion

Vancomycin-resistant enterococci have long been widespread in the community as well as in hospital settings [6,57,58,59,60]. New interventions against these pathogens are needed for prevention and treatment of resistant enterococcal infections. Phages constitute a safe and effective strategy against pathogenic bacteria. In this study, we were able to isolate two different phages from sewage samples. The genomic characterization of these phages indicated the absence of virulence, antibiotic resistance, and integrase genes, making them safe to be used in the larvae model and all in vivo models. The phages were able to eradicate multiple VRE*fm*, including daptomycin-resistant VRE*fm* strains isolated from different sources such as the environment, hospital rooms, and patients. Additionally, we showed the efficacy of a phage cocktail against this bacterium in vitro and in an in vivo larva model. Indeed, the VRE*fm*-specific phage cocktail composed of the two distinct phages was shown to be effective in a larvae model, reducing VRE*fm* abundance and increasing larval survival over a 48 h phage treatment compared to the group that did not receive phages but only VRE*fm*. Our results highlight the feasibility and the potential success of these phages in eradicating VRE*fm*.

Phage therapy is gaining more attention among the scientific and medical communities in Western countries for many reasons, including the slow process for new antibiotic development, and the increasing incidence of MDROs worldwide. Phages may present an approach that can potentially aid in the fight against MDRO colonization and infections. Naturally occurring phages with good safety profiles have been used in Western countries to treat infections caused by MDROs [20,22,23]. Nevertheless, there are a lack of in vivo VRE*fm*-specific phage studies performed on VRE*fm*-colonized and VRE*fm*-infected patients, including daptomycin-resistant VRE*fm*. 

The efficacy and safety of antimicrobial agents such as phages are assessed in an animal model before their potential application in humans. In vivo experiments are crucial to identify safety issues and the potential loss of activity due to host factors. However, experiments using murine models is time-consuming, expensive, and could be ethically objectionable. *Galleria mellonella* larvae or wax moth is an alternative and innovative model to study microbial virulence, as well as to evaluate the efficacy of antimicrobial agents such as antibiotics and phages [25,61,62]. It can serve as a pre-screening in vivo experiment preceding a mammalian model. This model is inexpensive, simple, and does not require ethical approval; moreover, *Galleria mellonella* survives at 37 °C, and has a significantly similar innate immune system to vertebrates [25,61,62,63]. The *Galleria mellonella* model has been adopted to evaluate phage therapy against multiple bacterial pathogens, demonstrating the efficacy of phages in vivo in increasing bacterial clearance and survival rates [24,64,65,66,67]. To our knowledge, no study has investigated this in vivo model to evaluate the efficacy of bacteriophages against VRE*fm*.

Few phages with activity against VRE*fm* have been isolated [68,69,70,71,72]. To our knowledge, two studies tested VRE*fm* phages in an in vivo model. Briefly, a single intraperitoneal injection of 3 × 10^8^ plaque-forming units of the VRE*fm* ENB6, isolated from raw sewage at a local municipal sewage treatment plant, administered 45 min after bacterial challenge with a clinical VRE*fm* isolate, was enough to rescue VRE*fm* bacteremic mice. In the absence of that phage, VRE*fm* bacteremia was fatal within 48 h [68]. A recent report showed the successful treatment of a VRE*fm* abdominal infection in a pediatric liver transplant patient that was failing many courses of antibiotics and surgical management. An individualized two-phage cocktail was administered intravenously over 2 h twice daily at a concentration of 8 × 10^7^ PFU/mL over a ten-day course of 2 mL/kg bodyweight followed by another ten days of 2 mL/kg bodyweight at a concentration of 5 × 10^8^ PFU/mL. To reduce the theoretical risk of an allergic reaction against the phage preparation, an H1-antagonist was administered before every phage application. A reduction in c-reactive protein starting the day after the first dose and constant clinical improvement were observed. There were no observed adverse events attributable to phage administration and VRE*fm* was not detected in the routine screening from rectal swabs [72].

## 5. Conclusions

Phages may constitute an alternative strategy to manage resistant bacteria such as VRE*fm* in an innovative, safe, and natural way. Our results highlight the feasibility and the potential success of these phages in inhibiting VRE*fm* in in vitro and in vivo models. Future studies in animal models as well as clinical trials to test the ability of these phages to safely and effectively eradicate VRE*fm*-colonized or infected animal and human hosts, such as immunocompromised cancer patients, in particular, are long overdue. In addition, the impact of phage therapy on the gut microbiota balance and the regulation of the immune system in these hosts should also be determined in future trials.

## Figures and Tables

**Table 1:** **Activity of phages against VRE*fm* isolated from different sources.** Boxes with (+) to show the ability of the phage to lyse the VRE*fm* strain tested, (-) indicates that the phage had no effect on the tested bacteria.

				Phages	
				MDA1	MDA2
Bacteria	Patient strains	Stool	VRE001	+	+
			VRE002	+	-
			VRE004	+	+
			VRE008	-	+
		Dapto-resistant	VRE33S	+	-
			VRE8S	+	-
	Environmental strains	Sewage	VREsewage1	+	-
			VREsewage2	+	-
		Rooms	VRE1147	+	+
			VRE1181	+	+
	Others		VSE	+	-
			*E. faecalis*	+	+

Abbreviations: VRE*fm*—vancomycin-resistant *Enterococcus faecium*; VSE—vancomycin-susceptible *Enterococcus faecium*; *E. faecalis*—vancomycin-susceptible *Enterococcus faecalis*; Dapto-resistant—daptomycin-resistant.

## Supplementary Materials

The following supporting information can be downloaded at: https://www.mdpi.com/article/10.3390/pharmaceutics14081591/s1, Supplementary Table S1: Gene characteristics of MDA1 genome; Supplementary Table S2: Gene characteristics of MDA2 genome; Figure S1: Genome comparison between MDA1 and Enterococcus phage vB_Efae230P-4. Figure S2: Genome comparison between MDA2 and Enterococcus phage phiEF24C.
