# Supplementary material for: Genomic and Functional Characterization of Vancomycin-Resistant Enterococci-Specific Bacteriophages in the Galleria mellonella Wax Moth Larvae Model
Source: Pharmaceutics. 2022 Jul 30;14(8):1591. doi: 10.3390/pharmaceutics14081591 (PMC9414631; doi:10.3390/pharmaceutics14081591)
Supplement: Supplementary file 1 [file pharmaceutics-14-01591-s001.zip › pharmaceutics-1832640-supplementary.pdf]

Supplementary Material: Genomic and Functional Characterization of Vancomycin Resistant Enterococci-specific Bacteriophages in the *Galleria mellonella* Wax Moth Larvae Model

Lynn El Haddad, Georgios Angelidakis, Justin R. Clark, Jesus F. Mendoza, Austen L. Terwilliger, Christopher Chaftari, Mark Duna, Serena T. Yusuf, Cynthia P. Harb, Mark Stibic , Anthony Maresso and Roy F. Chemaly

Table S1. Gene characteristics of MDA1 genome.

CDS	Start	End	Length	Direction	Function
**1**	444	629	185	Forward	Hypothetical protein
**2**	724	894	170	Forward	Hypothetical protein
**3**	896	2092	1196	Forward	Phage capsid and scaffold
**4**	2095	2343	248	Forward	Phage tail tube protein
**5**	2359	3390	1031	Forward	Phage capsid and scaffold
**6**	3323	3988	665	Forward	Hypothetical protein
**7**	4002	5561	1559	Forward	Hypothetical protein
**8**	5571	6365	794	Forward	Hypothetical protein
**9**	6406	8127	1721	Forward	Hypothetical protein
**10**	8127	8519	392	Forward	Hypothetical protein
**11**	8520	9296	776	Forward	N-acetylmuramoyl-L-alanine amidase
**12**	9612	9313	299	Reverse	Hypothetical protein
**13**	11149	9731	1418	Reverse	Hypothetical protein
**14**	11645	11142	503	Reverse	HNH homing endonuclease
**15**	11875	11642	233	Reverse	Hypothetical protein
**16**	12034	11876	158	Reverse	Hypothetical protein
**17**	12209	12036	173	Reverse	Hypothetical protein
**18**	12406	12200	206	Reverse	Hypothetical protein
**19**	12576	12406	170	Reverse	Hypothetical protein
**20**	14984	12636	2348	Reverse	Hypothetical protein
**21**	16237	14996	1241	Reverse	Phage neck
**22**	16652	16230	422	Reverse	Hypothetical protein
**23**	16829	16665	164	Reverse	Hypothetical protein
**24**	17229	16897	332	Reverse	Hypothetical protein
**25**	17917	17303	614	Reverse	Hypothetical protein

Table S2. Gene characteristics of MDA2 genome.

CDS	Type	Start	End	Length	Direction	Function
**1**	CDS	114	383	270	forward	lipoprotein
**2**	CDS	404	682	279	forward	membrane protein
**3**	CDS	686	1114	429	forward	Hypothetical protein
**4**	CDS	1114	1317	204	forward	Phage terminase, large subunit
**5**	CDS	1276	1383	108	reverse	Hypothetical protein
**6**	CDS	1497	1610	114	reverse	Hypothetical protein
**7**	CDS	1573	2538	966	forward	Hypothetical protein
**8**	CDS	2697	2789	93	forward	Phage terminase, large subunit
**9**	CDS	2936	3106	171	reverse	Hypothetical protein
**10**	CDS	3107	4579	1473	forward	Phage terminase, large subunit
**11**	CDS	4679	5467	789	forward	Hypothetical protein
**12**	CDS	5572	6288	717	forward	Hypothetical protein
**13**	CDS	6278	6622	345	forward	Hypothetical protein
**14**	CDS	6716	7585	870	forward	Phage N-acetylmuramoyl-L-alanine amidase
**15**	CDS	7752	8390	639	forward	Aggregation promoting factor peptidoglycan-binding protein
**16**	CDS	8532	8876	345	forward	Hypothetical protein

**17**	CDS	8954	10612	1659	forward	Portal protein
**18**	CDS	10622	10735	114	forward	Hypothetical protein
**19**	CDS	10707	11513	807	forward	Prohead protease protein
**20**	CDS	11520	12467	948	forward	Hypothetical protein
**21**	CDS	12608	14008	1401	forward	Phage major capsid protein
**22**	CDS	14107	14370	264	forward	Hypothetical protein
**23**	CDS	14383	15282	900	forward	Prohead protein
**24**	CDS	15299	16168	870	forward	Head completion protein
**25**	CDS	16161	16784	624	forward	Hypothetical protein
**26**	CDS	16788	17633	846	forward	Baseplate assembly protein
**27**	CDS	17633	17866	234	forward	Hypothetical protein
**28**	CDS	17870	19579	1710	forward	Phage tail sheath CDS
**29**	CDS	19640	20062	423	forward	Tail tube protein
**30**	CDS	20154	21251	1098	forward	Endonuclease
**31**	CDS	21248	21394	147	forward	Hypothetical protein
**32**	CDS	21530	22003	474	forward	Tail tape measure protein
**33**	CDS	22071	22646	576	forward	RNA polymerase
**34**	CDS	22691	26347	3657	forward	Tail tape measure
**35**	CDS	26386	29571	3186	forward	Secretory antigen SsaA-like protein - putative tail lysin
**36**	CDS	29657	35134	5478	forward	Glycerophosphoryl diester phosphodiesterase tail fiber protein
**37**	CDS	35234	37645	2412	forward	Phage capsid and scaffold
**38**	CDS	37639	38379	741	forward	Hypothetical protein
**39**	CDS	38396	38542	147	forward	Hypothetical protein
**40**	CDS	38617	38712	96	forward	Hypothetical protein
**41**	CDS	38679	39368	690	forward	Hypothetical protein
**42**	CDS	39372	39908	537	forward	Hypothetical protein
**43**	CDS	39895	40599	705	forward	Baseplate assembly protein
**44**	CDS	40615	41667	103	forward	Baseplate
**45**	CDS	41686	43086	1401	forward	Hypothetical protein
**46**	CDS	43083	43211	129	reverse	Hypothetical protein
**47**	CDS	43258	43737	480	forward	Hypothetical protein
**48**	CDS	43752	47216	3465	forward	Adsorption tail protein
**49**	CDS	47295	47501	207	forward	Hypothetical protein
**50**	CDS	47756	49528	1773	forward	DNA helicase
**51**	CDS	49556	51184	1629	forward	Transcriptional regulator
**52**	CDS	51217	52689	1473	forward	DNA helicase
**53**	CDS	52689	53744	1056	forward	Recombination exonuclease
**54**	CDS	53860	>55916	>2057	forward	Recombination related exonuclease
**55**	CDS	55921	56586	666	forward	Hypothetical protein
**56**	CDS	56587	57645	1059	forward	DNA primase/helicase
**57**	CDS	57662	58294	633	forward	Hypothetical protein
**58**	CDS	58320	59204	885	forward	Hypothetical protein
**59**	CDS	59207	59437	231	forward	Hypothetical protein
**60**	CDS	59439	59747	309	forward	Hypothetical protein
**61**	CDS	59734	60045	312	forward	Hypothetical protein
**62**	CDS	60038	60409	372	forward	Hypothetical protein
**63**	CDS	60429	61097	669	forward	Hypothetical protein
**64**	CDS	61099	61398	300	forward	Hypothetical protein
**65**	CDS	61404	61883	480	forward	Hypothetical protein
**66**	CDS	61976	62770	795	forward	Hypothetical protein
**67**	CDS	62763	63074	312	forward	Phage integration host factor
**68**	CDS	63164	65566	2403	forward	DNA polymerase I
**69**	CDS	65705	65803	99	reverse	Hypothetical protein
**70**	CDS	66006	66095	90	reverse	Hypothetical protein
**71**	CDS	66116	66220	105	reverse	Hypothetical protein
**72**	CDS	66195	66674	480	forward	DNA polymerase I
**73**	CDS	66777	67319	543	forward	Hypothetical protein

**74**	CDS	67374	68651	1278	forward	Hypothetical protein
**75**	CDS	68736	69983	1248	forward	Phage recombinase
**76**	CDS	70109	70423	315	forward	Hypothetical protein
**77**	CDS	70416	71030	615	forward	Phage holin
**78**	CDS	71091	71366	276	forward	Hypothetical protein
**79**	CDS	71414	72376	963	forward	Hypothetical protein
**80**	CDS	72397	72840	444	forward	Hypothetical protein
**81**	CDS	72947	73252	306	forward	Hypothetical protein
**82**	CDS	73249	74202	954	forward	Hypothetical protein
**83**	CDS	74256	75539	1284	forward	Hypothetical protein
**84**	CDS	75551	75925	375	forward	Hypothetical protein
**85**	CDS	75964	76584	621	forward	Hypothetical protein
**86**	CDS	76584	77324	741	forward	Hypothetical protein
**87**	CDS	77314	77820	507	forward	Hypothetical protein
**88**	CDS	77834	78184	351	forward	Hypothetical protein
**89**	CDS	78209	79066	858	forward	Hypothetical protein
**90**	CDS	79170	79583	414	forward	Thioredoxin, phage-associated
**91**	CDS	79489	79971	483	forward	Thioredoxin, phage-associated
**92**	CDS	79964	81601	1638	forward	Hypothetical protein
**93**	CDS	81766	81864	99	forward	Hypothetical protein
**94**	CDS	81911	82600	690	forward	Hypothetical protein
**95**	CDS	82611	83078	468	forward	Hypothetical protein
**96**	CDS	83179	85452	2274	forward	Hypothetical protein
**97**	CDS	85542	85718	177	forward	Hypothetical protein
**98**	CDS	85740	86246	507	forward	Hypothetical protein
**99**	CDS	86320	86490	171	forward	Hypothetical protein
**100**	CDS	86480	86755	276	forward	Hypothetical protein
**101**	CDS	86863	87114	252	forward	Hypothetical protein
**102**	CDS	87126	87413	288	forward	Hypothetical protein
**103**	CDS	87416	88204	789	forward	Hypothetical protein
**104**	CDS	88287	89360	1074	forward	Hypothetical protein
**105**	CDS	89485	89787	303	forward	Hypothetical protein
**106**	CDS	89789	90091	303	forward	Hypothetical protein
**107**	CDS	90094	90387	294	forward	Hypothetical protein
**108**	CDS	90384	90563	180	forward	Hypothetical protein
**109**	CDS	90576	90926	351	forward	Hypothetical protein
**110**	CDS	90953	91330	378	forward	Hypothetical protein
**111**	CDS	91323	91553	231	forward	Hypothetical protein
**112**	CDS	91557	91931	375	forward	Hypothetical protein
**113**	CDS	91928	92170	243	forward	Hypothetical protein
**114**	CDS	92188	92376	189	forward	Hypothetical protein
**115**	CDS	92373	92972	600	forward	Hypothetical protein
**116**	CDS	93014	93856	843	forward	Hypothetical protein
**117**	CDS	93869	94498	630	forward	Hypothetical protein
**118**	CDS	94576	94818	243	forward	Hypothetical protein
**119**	CDS	94836	94976	141	forward	Hypothetical protein
**120**	CDS	94989	95321	333	forward	Hypothetical protein
**121**	CDS	95386	96027	642	forward	Hypothetical protein
**122**	CDS	96040	96207	168	forward	Hypothetical protein
**123**	CDS	96305	96634	330	forward	Hypothetical protein
**124**	CDS	96802	97233	432	forward	Hypothetical protein
**125**	CDS	97277	97375	99	forward	Hypothetical protein
**126**	CDS	97372	97503	132	reverse	Hypothetical protein
**127**	CDS	97574	97873	300	forward	Hypothetical protein
**128**	CDS	97952	98275	324	forward	Hypothetical protein
**129**	CDS	98372	98512	141	forward	Hypothetical protein
**130**	CDS	98571	98798	228	forward	Hypothetical protein
**131**	CDS	98880	99173	294	forward	Hypothetical protein

**132**	CDS	99284	99490	207	forward	Hypothetical protein
**133**	CDS	99572	99814	243	forward	Hypothetical protein
**134**	CDS	99891	100136	246	forward	Hypothetical protein
**135**	CDS	100210	100416	207	forward	Hypothetical protein
**136**	CDS	100383	100535	153	reverse	Hypothetical protein
**137**	CDS	100597	100767	171	forward	Hypothetical protein
**138**	CDS	100838	101134	297	forward	Hypothetical protein
**139**	CDS	101204	101401	198	forward	Hypothetical protein
**140**	CDS	101395	101598	204	forward	Hypothetical protein
**141**	CDS	101604	101915	312	forward	Hypothetical protein
**142**	CDS	101903	102094	192	forward	Hypothetical protein
**143**	CDS	102107	102466	360	forward	Hypothetical protein
**144**	CDS	102471	102572	102	forward	Hypothetical protein
**145**	CDS	103332	103433	102	reverse	Hypothetical protein
**146**	CDS	103417	103521	105	reverse	Hypothetical protein
**147**	CDS	103530	103688	159	reverse	Hypothetical protein
**148**	CDS	103725	103970	246	forward	Hypothetical protein
**149**	CDS	104086	104412	327	forward	Hypothetical protein
**150**	CDS	104485	104709	225	forward	Hypothetical protein
**151**	CDS	104811	104984	174	forward	Hypothetical protein
**152**	CDS	105087	105782	696	forward	Hypothetical protein
**153**	CDS	105927	106025	99	reverse	Hypothetical protein
**154**	CDS	106012	106155	144	forward	Hypothetical protein
**155**	CDS	106235	106354	120	forward	Hypothetical protein
**156**	CDS	106452	106679	228	reverse	Hypothetical protein
**157**	CDS	106681	106980	300	reverse	Hypothetical protein
**158**	CDS	107003	107314	312	reverse	Hypothetical protein
**159**	CDS	107370	107681	312	reverse	Hypothetical protein
**160**	CDS	107716	107943	228	reverse	Hypothetical protein
**161**	CDS	107944	108249	306	reverse	Hypothetical protein
**162**	CDS	108422	108613	192	reverse	Hypothetical protein
**163**	CDS	108657	108881	225	reverse	Hypothetical protein
**164**	CDS	108895	109146	252	reverse	Hypothetical protein
**165**	CDS	109147	109527	381	reverse	Hypothetical protein
**166**	CDS	109540	109698	159	reverse	Hypothetical protein
**167**	CDS	109755	109952	198	reverse	Hypothetical protein
**168**	CDS	109952	110467	516	reverse	Phage protein (ACLAME 82)
**169**	CDS	110480	110728	249	reverse	Hypothetical protein
**170**	CDS	110741	111283	543	reverse	Hypothetical protein
**171**	CDS	111284	111676	393	reverse	Hypothetical protein
**172**	CDS	111669	112028	360	reverse	Hypothetical protein
**173**	CDS	112032	112202	171	reverse	Hypothetical protein
**174**	CDS	112190	112732	543	reverse	Exonuclease
**175**	CDS	112735	113199	465	reverse	Hypothetical protein
**176**	CDS	113196	113666	471	reverse	Hypothetical protein
**177**	CDS	113682	113882	201	reverse	Hypothetical protein
**178**	CDS	113996	114361	366	reverse	Hypothetical protein
**179**	CDS	114358	114537	180	reverse	Hypothetical protein
**180**	CDS	114551	115069	519	reverse	Uncharacterized protein COG3236
**181**	CDS	115070	115543	474	reverse	Hypothetical protein
**182**	CDS	115540	116250	711	reverse	Hypothetical protein
**183**	CDS	116276	116716	441	reverse	Hypothetical protein
**184**	CDS	116766	116954	189	reverse	Hypothetical protein
**185**	CDS	116951	117412	462	reverse	Hypothetical protein
**186**	CDS	117426	117602	177	reverse	Hypothetical protein
**187**	CDS	117599	117796	198	reverse	Hypothetical protein
**188**	CDS	117793	118230	438	reverse	Hypothetical protein
**189**	CDS	118321	119004	684	reverse	Serine/threonine protein phosphatase

**190**	CDS	119058	119537	480	reverse	Hypothetical protein
**191**	CDS	119541	119792	252	reverse	Hypothetical protein
**192**	CDS	119789	120400	612	reverse	Phosphoesterase
**193**	CDS	120413	120730	318	reverse	Hypothetical protein
**194**	CDS	120760	120876	117	reverse	Hypothetical protein
**195**	CDS	120890	121102	213	reverse	Hypothetical protein
**196**	CDS	121183	121596	414	reverse	Hypothetical protein
**197**	CDS	121601	121984	384	reverse	Hypothetical protein
**198**	CDS	121968	122261	294	reverse	Hypothetical protein
**199**	CDS	122224	122451	228	reverse	Hypothetical protein
**200**	CDS	122507	122632	126	reverse	Hypothetical protein
**201**	CDS	122708	123052	345	reverse	putative adsorption protein
**202**	CDS	123052	123306	255	reverse	Hypothetical protein
**203**	CDS	123287	123541	255	reverse	Hypothetical protein
**204**	CDS	123552	123887	336	reverse	Hypothetical protein
**205**	CDS	123862	124299	438	reverse	Hypothetical protein
**206**	CDS	124315	124776	462	reverse	Hypothetical protein
**207**	CDS	124769	124996	228	reverse	Hypothetical protein
**208**	CDS	124984	125415	432	reverse	Hypothetical protein
**209**	CDS	125418	125648	231	reverse	Hypothetical protein
**210**	CDS	125645	126076	432	reverse	Hypothetical protein
**211**	CDS	126080	126232	153	reverse	Hypothetical protein
**212**	CDS	126232	127179	948	reverse	Thymidylate synthase
**213**	CDS	127229	127615	387	reverse	Hypothetical protein
**214**	CDS	127709	127858	150	reverse	Hypothetical protein
**215**	CDS	127858	128715	858	reverse	Hypothetical protein
**216**	CDS	128763	128864	102	reverse	Hypothetical protein
**217**	CDS	128877	129860	984	reverse	Ribonucleotide reductase of class Ib, beta subunit
**218**	CDS	129873	132023	2151	reverse	Ribonucleotide reductase of class Ib, alpha subunit
**219**	CDS	132026	132268	243	reverse	Glutaredoxin-like protein NrdH, required for reduction of Ribonucleotide reductase class Ib
**220**	CDS	132397	132603	207	reverse	Hypothetical protein
**221**	CDS	132669	132974	306	reverse	Hypothetical protein
**222**	CDS	133065	133295	231	reverse	Hypothetical protein
**223**	CDS	133359	133583	225	reverse	Hypothetical protein
**224**	CDS	133694	133978	285	reverse	Hypothetical protein
**-**	tRNA1	134030	134101	72	reverse	tRNA-Thr-TGT
**225**	CDS	134127	135218	1092	reverse	Hypothetical protein
**226**	CDS	135285	135440	156	reverse	Hypothetical protein
**-**	tRNA2	135589	135662	74	reverse	tRNA-Met-CAT
**-**	tRNA3	135721	135794	74	reverse	tRNA-Pro-TGG
**227**	CDS	136108	136254	147	reverse	Hypothetical protein
**228**	CDS	136259	136366	108	forward	Hypothetical protein
**229**	CDS	136431	136553	123	forward	Hypothetical protein
**-**	tRNA4	136744	136817	74	reverse	tRNA-Arg-TCT
**230**	CDS	137029	137157	129	forward	Hypothetical protein
**231**	CDS	137112	137216	105	reverse	Hypothetical protein
**-**	tRNA5	137303	137374	72	reverse	tRNA-Trp-CCA
**232**	CDS	137360	137482	123	reverse	Hypothetical protein
**233**	CDS	137537	137662	126	reverse	Hypothetical protein
**-**	tRNA6	137629	137701	73	reverse	tRNA-Pseudo-GTC
**234**	CDS	137654	137746	93	forward	Hypothetical protein
**235**	CDS	138042	138143	102	forward	Hypothetical protein
**236**	CDS	138100	138222	123	forward	Hypothetical protein
**237**	CDS	138373	138495	123	reverse	Hypothetical protein
**238**	CDS	138540	138695	156		Hypothetical protein
**239**	CDS	138748	138843	96		Hypothetical protein
**240**	CDS	139182	139520	339		Hypothetical protein

**241**	CDS	139584	139928	345	Hypothetical protein
**242**	CDS	139963	140223	261	Hypothetical protein

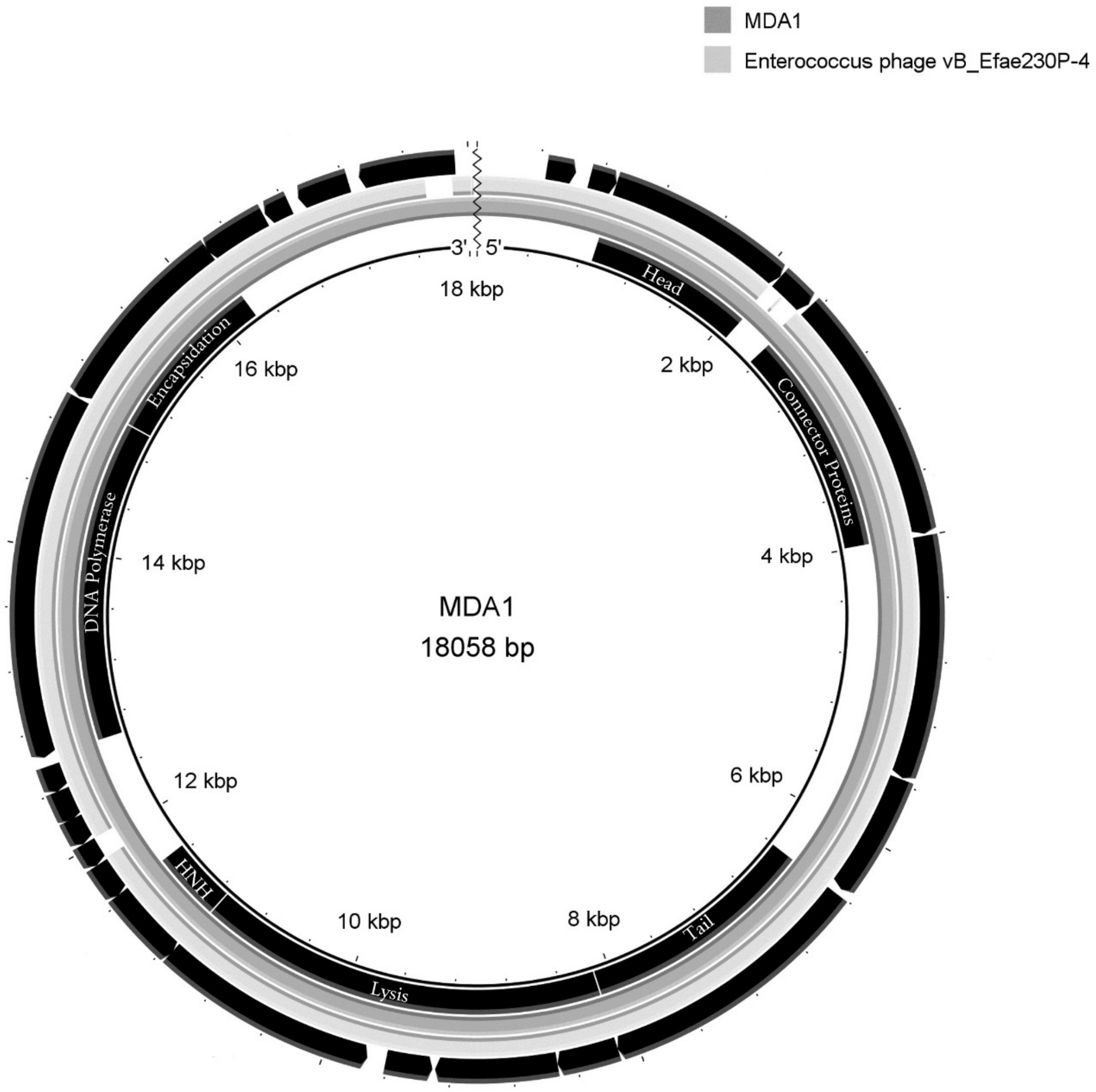

Figure S1. Genome comparison between MDA1 and Enterococcus phage vB_Efae230P-4.

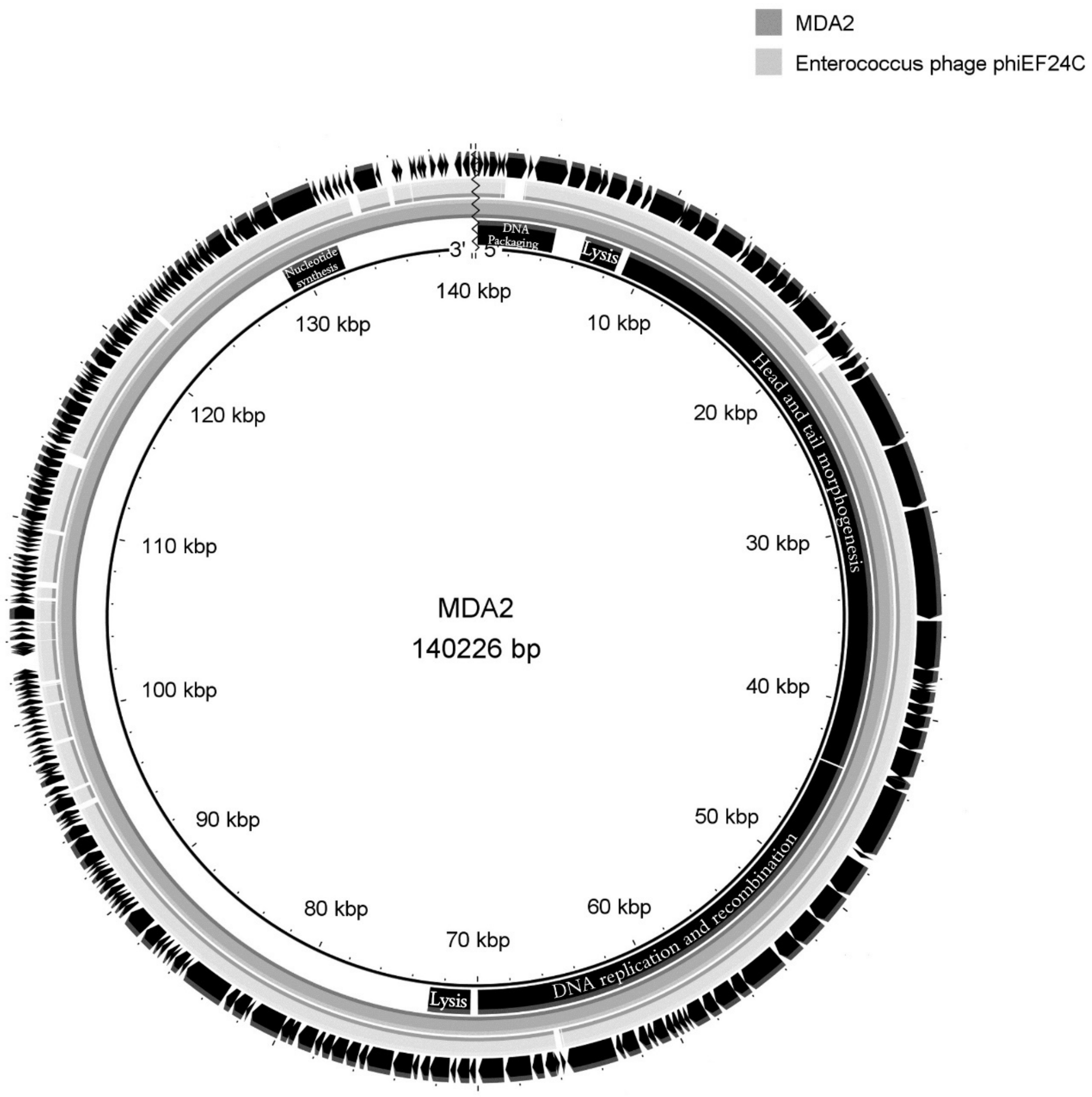

Figure S2. Genome comparison between MDA2 and Enterococcus phage phiEF24C.
